# Physiological Integration Increases Sexual Reproductive Performance of the Rhizomatous Grass *Hierochloe glabra*

**DOI:** 10.3390/plants9111608

**Published:** 2020-11-19

**Authors:** Jian Guo, Haiyan Li, Yunfei Yang

**Affiliations:** Key Laboratory of Vegetation Ecology, Ministry of Education, Institute of Grassland Science, Northeast Normal University, Changchun 130024, China; guoj481@nenu.edu.cn

**Keywords:** companion species, perennial herb, resource translocation, sexual reproduction, tillering node, vegetative ramet

## Abstract

Clonal plants usually reproduce asexually through vegetative propagation and sexually by producing seeds. Physiological integration, the translocation of essential resources between ramets, usually improves vegetative reproduction. However, how physiological integration affects sexual reproduction has been less studied in clonal grasses. Here, we chose *Hierochloe glabra*, a major early spring forage of the eastern Eurasian steppe, and conducted a series of field experiments, including sampling reproductive ramets connected by tillering nodes to different numbers of vegetative ramets and ^15^N leaf labeling of ramet pairs at the seed-filling stage. In the natural populations of *H. glabra*, vegetative ramets were taller, had more and larger leaves, and greater biomass than reproductive ramets. Except for reproductive ramet biomass, sexual reproductive characteristics significantly increased with an increase in the number and biomass of vegetative ramets connected to tillering nodes. ^15^N labeling showed that vegetative ramets supplied nutrients to reproductive ramets through tillering nodes. Overall, our results indicate that significant differences in morphological characteristics and biomass allocation underlie resources translocation from vegetative ramets towards reproductive ramets. Physiological integration between different functional ramets can increase sexual reproductive performance, which will be beneficial to population persistence in *H. glabra*.

## 1. Introduction

Environments are generally heterogeneous, and the availability of essential resources needed for plant growth, such as water, light, and nutrients, is commonly changeable at small spatial scales [1]. In grassland plant communities, many plant species are clonal organisms, and one of their strategies for coping with this environmental heterogeneity is physiological integration [2,3,4]. Physiological integration refers to the translocation and sharing of essential resources, such as water, mineral nutrients, and carbohydrates, between ramets through physical connections in the same individual [5,6]. Numerous studies have demonstrated that physiological integration can promote the establishment of newly generated ramets [7,8,9], help genets expand into open spaces [10], and improve the survival and performance of ramets in heterogeneous environments [2,4,11,12,13,14].

Clonal plants can produce not only genetically identical and potentially physiologically independent individuals (called ramets) through vegetative propagation but also seeds by means of sexual reproduction [15]. Some clonal plants are often thought to primarily rely on vegetative propagation to achieve natural population regeneration with infrequent recruitment from seeds [16,17], but sexual reproduction through seeds produces genetically diverse offspring that may be greatly beneficial in changing environments [18]. Moreover, sexual reproduction also plays an important role in the long-distance dispersal of offspring and may thus reduce local intraspecific competition and aid in the colonization of new habitats [19]. Therefore, even for clonal plants in different environments, sexual reproduction is still very important for population persistence. In fact, studies concerning the effects of physiological integration between connected ramets on reproductive performance are mostly conducted through pot experiments in greenhouses [4,12]; because of limited growth time, growth space, and the microenvironment, sexual reproduction rarely occurs in pot experiments. Accordingly, the majority of these studies reveal the effect of physiological integration on vegetative propagative performance, and only a few studies reveal the effect on the sexual reproductive performance of clonal plants [20,21]. Field experiments can provide a more realistic test compared to pot experiments in greenhouses. Thus, it appears that it will be of great significance to carry out research on the effect of physiological integration on the sexual reproductive performance of clonal plants in the field.

From the perspective of ramet function (rather than the developmental age of the ramet), two different types of aboveground ramets are widely distributed in clonal plant populations: vegetative ramets and reproductive ramets [22], and they can be interconnected by stolons, rhizomes, or tillering nodes. Thus, the so-called paired ramet system often includes the following three cases: vegetative ramet-vegetative ramet, reproductive ramet-reproductive ramet, and vegetative ramet-reproductive ramet; and the first two cases are paired ramet systems with the same function. Surprisingly, in the study of physiological integration, a large body of attention has been given to the integration between vegetative ramet and vegetative ramet connected by the stolon or rhizome [2,4,5,9,11]; so far, the integration between vegetative ramet and reproductive ramet connected by the tillering node, in particular, has been less studied. Direct tests of the effect of vegetative ramets connected by tillering nodes to reproductive ramets on reproductive ramets appear to have focused mainly on cereal crops. Vegetative ramets are thought to compete with reproductive ramets for solar energy, carbohydrates, and mineral nutrients, for example, nitrogen in rice [23]; thus, an increase in vegetative ramets may cause fewer nutrients to be directed to grain production. Nevertheless, Ao et al. found that an increase in vegetative ramets did not reduce the grain yield of rice [24]. Similarly, the evident inconsistencies in the effects of vegetative ramets on grain production have also been observed in wheat [25,26]. However, it is not well known how physiological integration between vegetative ramet and reproductive ramet connected by the tillering node affects seed production in clonal grasses.

Clonal grasses may be dominant species or companion species in plant communities. Dominant species are species that have a high abundance relative to other species in a community and have proportionate effects on community structure and environmental conditions [27], while companion species are species that occur frequently in the community but do not have major effects on community structure and environmental conditions [28]. Semiarid and arid grasslands in the eastern part of Eurasia are an important part of the world’s grassland biota. Perennial rhizomatous *Leymus chinensis* is one of the main dominant species of climax plant communities, and perennial rhizomatous *Hierochloe glabra* is one of the main companion species [29]. Compared with *L. chinensis*, *H. glabra* is shorter, begins turning green earlier, and has vigorous vegetative propagation, and it often exhibits explosive population growth under conditions with enough growth space and without interspecific competition [30]. Previous studies regarding *H. glabra* have mainly focused on its vegetative propagative characteristics [30], spatial expansion strategies [31], and epigenetics and adaptation [32]. Thus far, physiological integration between connected ramets has not been reported. Although companion species are not as important as dominant species, they are important indicators of the community succession process. Therefore, a study on physiological integration in companion species will help provide a comprehensive understanding of their adaptation and status in the community.

Here, we used *H. glabra*, a common companion species of plant communities in the eastern Eurasian steppe, as the study species. In the natural populations of *H. glabra*, we investigated the morphological characteristics and biomass allocation patterns of both reproductive and vegetative ramets and determined the sexual reproductive characteristics when connected to different numbers of vegetative ramets. We also labeled the vegetative ramets with an isotope (^15^N) at the seed-filling stage to verify whether vegetative ramets translocated resources towards the connected reproductive ramets. The objectives of our study were (1) to explore the differences in morphological characteristics and biomass allocation between reproductive ramets and vegetative ramets and (2) to assess the effect of physiological integration on sexual reproductive performance. We hypothesize that (1) vegetative ramets will have more leaves, a larger leaf area, and greater leaf biomass, and (2) physiological integration will increase sexual reproductive performance.

## 2. Results

### 2.1. Morphological Characteristics

At the seed-maturation stage of *H. glabra*, vegetative ramets were significantly taller than reproductive ramets (Table 1, Supplementary Table S1). The reproductive ramets consisted of two leaves; the second leaf had a significantly larger length, width, and area than those of the first leaf (Table 1, Supplementary Table S2), and the average area of the second leaf (0.43 cm^2^, accounting for 68.3% of the total leaf area) was 2.2 times as large as that of the first leaf (0.20 cm^2^, accounting for 31.7% of the total leaf area). The vegetative ramets consisted of four leaves. Leaf position significantly affected the leaf length, width, and area of vegetative ramets (Table 1, Supplementary Table S3). With an increase in leaf position, the leaf length, width, and area all significantly decreased (Table 1). The total leaf area of vegetative ramets was significantly larger than that of reproductive ramets (Table 1, Supplementary Table S1).

### 2.2. Biomass Allocation

At the seed-maturation stage of *H. glabra*, there were significant effects from organ type on the biomass and allocation percentage of reproductive ramets (Supplementary Table S4). The biomass and allocation percentage of the panicle were both significantly greater than those of the other three organs, the biomass and allocation percentage of the leaf were both significantly smaller than those of the other three organs, and the panicle biomass was greater than the leaf biomass by 3.4 times (Table 2). For vegetative ramets, the leaf had significantly greater biomass and allocation percentage than those of the sheath (Supplementary Table S5), and the leaf biomass was greater than the sheath biomass by 0.6 times. The total biomass of vegetative ramets was significantly greater than that of reproductive ramets (Table 2, Supplementary Table S1).

### 2.3. Sexual Reproductive Characteristics

**Figure 1:**
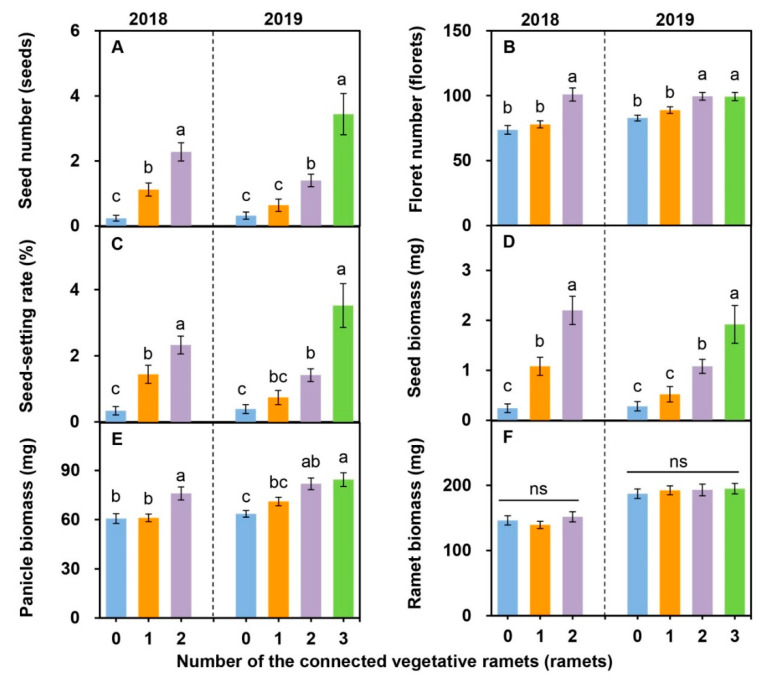
Effects of the number of vegetative ramets connected to tillering nodes on sexual reproductive characteristics (seed number, (**A**); floret number, (**B**); seed-setting rate, (**C**); seed biomass, (**D**); panicle biomass, (**E**); ramet biomass, (**F**)) in the natural populations of *Hierochloe glabra* over two consecutive years (data are represented as the means ± standard errors, *n* = 25). Different lowercase letters indicate significant differences among different numbers of vegetative ramets (*p* < 0.05); ns represents that there is no significant difference between different numbers of connecting vegetative ramets (*p* > 0.05).

The number of vegetative ramets connected to tillering nodes had significant effects on the seed number (Figure 1A), floret number (Figure 1B), seed-setting rate (Figure 1C), seed biomass (Figure 1D), and panicle biomass (Figure 1E), but not on the ramet biomass (Figure 1F) (Supplementary Table S6). With an increase in the number of vegetative ramets connected to tillering nodes, the seed number, floret number, seed-setting rate, seed biomass, and panicle biomass showed increasing trends (Figure 1A–E). The seed number, floret number, seed-setting rate, seed biomass, and panicle biomass of the reproductive ramets connected to ≥ two vegetative ramets were significantly greater than those of the reproductive ramets connected to zero vegetative ramets (Figure 1A–E).

### 2.4. Relationships between Soil Physicochemical Properties and Sexual Reproductive Characteristics as Well as Growth Characteristics of Vegetative Ramets

There were little differences in the soil moisture, soil bulk density, soil pH, electric conductivity, total organic C concentration, total N concentration, and total P concentration among the twenty-five clones of *H. glabra* (Supplementary Table S7), indicating that the soil physicochemical properties of these *H. glabra* clones were very similar. The reproductive ramet biomass was significantly and negatively correlated with soil moisture, while the seed number, floret number, seed-setting rate, seed biomass, panicle biomass, the leaf biomass of vegetative ramet, and vegetative ramet biomass were not significantly correlated with soil physicochemical properties (Table 3). These results indicated that soil physicochemical properties basically neither affected the sexual reproductive characteristics nor affected the growth characteristics of vegetative ramets in the natural populations of *H. glabra*.

### 2.5. Relationships between Sexual Reproductive Characteristics and the Leaf Biomass and Total Biomass of Vegetative Ramets Connected to Tillering Nodes

**Figure 2:**
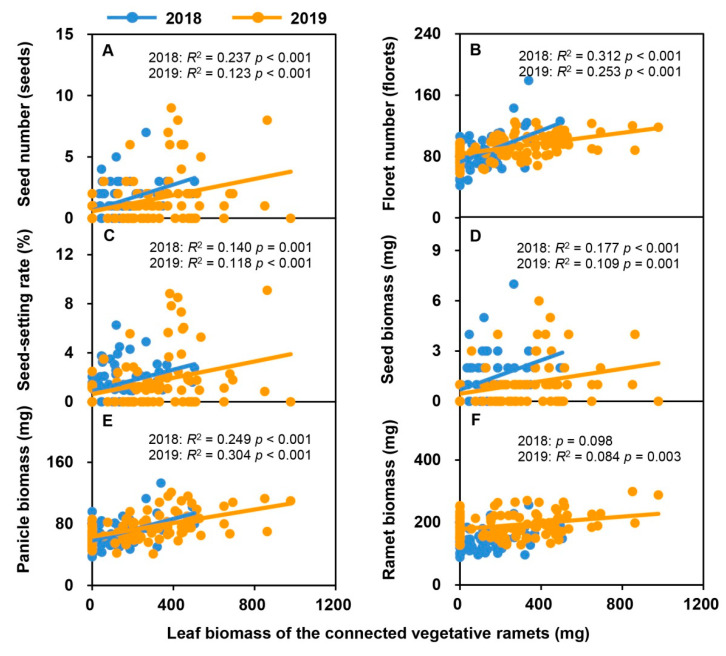
Relationships between sexual reproductive characteristics (seed number, (**A**); floret number, (**B**); seed-setting rate, (**C**); seed biomass, (**D**); panicle biomass, (**E**); ramet biomass, (**F**)) and leaf biomass of vegetative ramets connected to tillering nodes in the natural populations of *Hierochloe glabra* over two consecutive years. The colored lines represent fitting lines in 2018 (*n* = 75) and 2019 (*n* = 100).

**Figure 3:**
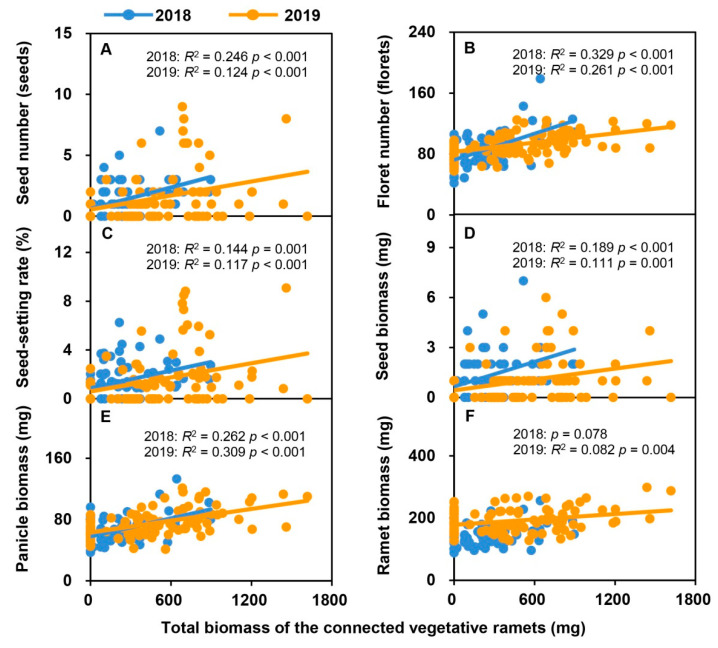
Relationships between sexual reproductive characteristics (seed number, (**A**); floret number, (**B**); seed-setting rate, (**C**); seed biomass, (**D**); panicle biomass, (**E**); ramet biomass, (**F**)) and total biomass of vegetative ramets connected to tillering nodes in the natural populations of *Hierochloe glabra* over two consecutive years. The colored lines represent fitting lines in 2018 (*n* = 75) and 2019 (*n* = 100).

With an increase in the leaf biomass and total biomass of vegetative ramets connected to tillering nodes over the two consecutive years, the seed number, floret number, seed-setting rate, seed biomass, panicle biomass, and reproductive ramet biomass of the *H. glabra* population increased linearly (Figure 2 and Figure 3). Except for the ramet biomass in 2018, all other relationships reached a significant level (Figure 2A–F and Figure 3A–F).

### 2.6. Transfer of ^15^N among Ramets

**Figure 4:**
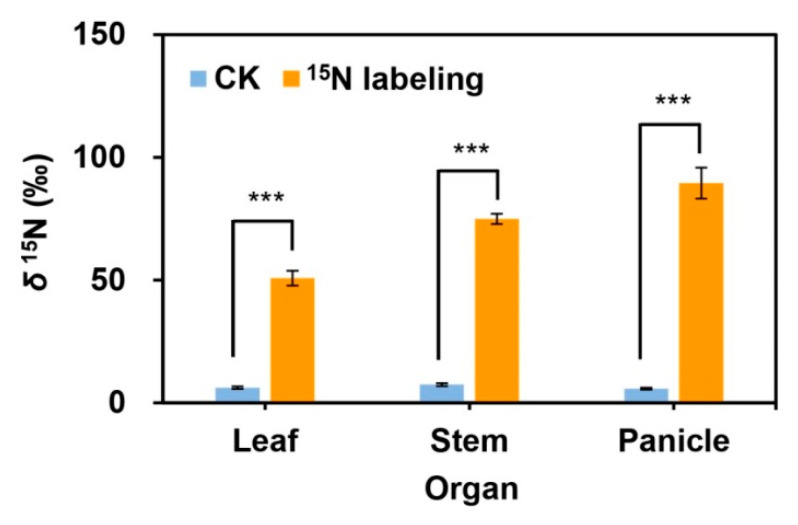
Comparison of the leaf *δ*^15^N, stem *δ*^15^N, and panicle *δ*^15^N of reproductive ramets between the control and ^15^N labeling treatments in the natural populations of *Hierochloe glabra* (data are represented as the means ± standard errors, *n* = 5). ***, *p* < 0.001.

Reproductive ramets had significantly greater leaf *δ*^15^N, stem *δ*^15^N, and panicle *δ*^15^N in the ^15^N labeling treatment than in the control treatment (no ^15^N labeling) (Figure 4, Supplementary Table S8). These results demonstrated that vegetative ramets connected to tillering nodes transferred nutrients towards reproductive ramets through physiological integration.

## 3. Discussion

### 3.1. Biomass Allocation

Allocation of biomass to various organs is a core component of plant life history [33,34]. In general, total resources are limited in plants, and increasing resource allocation to one function or organ may lead to a decreasing allocation to other functions or organs [33,35]. Once a plant initiates its reproductive machinery through growth, its biomass allocation requires investment trade-offs [36]. At the seed-maturation stage of *H. glabra*, individual reproductive ramet allocated 34.29% of its total biomass to the panicle for reproduction and 65.71% to the leaves, sheath, and stem for vegetative growth, but the leaf allocation percentage was the smallest, at only 7.88% (Table 2). As the major photosynthetic organs, leaves can export nutrients and are the most important source organs, while panicles require large amounts of nutrients and are sink organs. The allocation of biomass between source and sink organs is the major determinant of plant growth and productivity [37]. The low leaf allocation percentage in the reproductive ramets of *H. glabra* may reflect a serious shortage of nutrients supplied from the source organs to the sink organs, implying that the growth of reproductive ramets themselves likely requires the supply of external nutrients. Under natural conditions, compared with reproductive ramets, vegetative ramets were taller, had more and larger leaves (Table 1), and greater leaf biomass (Table 2), just as the first hypothesis we put forward, thereby increasing light interception and photosynthesis.

Compared with vegetative propagation, sexual reproduction is considered to consume more resources and thus to be more nutrient demanding [38]. For example, the mean biomass of reproductive ramets was nearly twice that of vegetative ramets in *Iris laevigata* [39]. However, we found that the mean biomass of reproductive ramets was 27.2% that of vegetative ramets in *H. glabra* (Table 2). This result suggests that the total resources of vegetative ramets are much greater than reproductive ramets, and vegetative ramets likely will provide nutrients for their connected reproductive ramets so that reproductive ramets can better perform a reproductive function.

### 3.2. Vegetative Ramets Connected to Tillering Nodes Positively Affect Sexual Reproductive Performance

In clonal grass populations, it is often seen that a reproductive ramet has different numbers of connecting vegetative ramets on its tillering nodes. From the perspective of reproductive ramets themselves, this is possibly because there are differences in size and lifespan of reproductive ramets or capacity for vegetative propagation of tillering nodes of reproductive ramets [40,41]. In the present study, at the early heading stage, we selected reproductive ramets of a similar size and with different numbers of connecting vegetative ramets at the edge of each clone as the experimental samples for tagging. After approximately thirty days of growth, when the seeds were mature, we found that there was no significant difference in the biomass of reproductive ramets among different numbers of connecting vegetative ramets (Figure 1F), so was the height of reproductive ramets (data not shown), which suggested that the difference in the number of connecting vegetative ramets did not result from the difference in the size of reproductive ramets. A number of studies have shown that with regard to the perennial fleshy rhizomatous *H. glabra*, the lifespan of tillering nodes and rhizomes is three years at most [42]. So, in the natural populations of *H. glabra*, usual growth patterns of tillering nodes are as follows: in the first year of life, during the growing season, tillering nodes, which are derived from clonal fragments and almost unlikely from seedlings, produce long belowground rhizomes [43]; a large number of buds and rosette juvenile ramets are formed at the rhizome tips at the end of the growing season [44]; in the second year of life, reproductive ramets appear and are totally grown from overwintering juvenile ramets [43], and different numbers of vegetative ramets on the tillering nodes of reproductive ramets are grown from overwintering juvenile ramets or buds; in the third year of life, very few ramets appear, and they are impossible to flower. This suggests that reproductive ramets of *H. glabra* are all formed in the second year of life, and they have an identical lifespan. Therefore, the difference in the number of connecting vegetative ramets involved in this study is more likely due to the difference in capacity for vegetative propagation of tillering nodes of reproductive ramets and, consequently, the number of axillary buds produced.

Most perennial clonal plants possess the capacity for sexual reproduction by seeds and clonal reproduction through vegetative propagules [15]. It is often thought that sexual reproduction and clonal reproduction compete for resources [45], and thus, under the condition of limited resources, increased investment in sexual reproduction will result in a decrease investment in clonal reproduction and vice versa [46]. For example, investment of resources in seed has substantially decreased clonal propagule production of *Butomus umbellatus* [47] and *Sagittaria latifolia* [48]. In addition, sexual and clonal reproduction may also be dependent upon different limiting resources, so increased investment in sexual reproduction will not necessarily lead to a proportional decline in clonal reproduction [49]. For instance, resource investment in male function would not decrease clonal propagule production of *S. latifolia*, whereas it has decreased the nutrient content of clonal propagules [48]. In the present study, we found a significant positive correlation between five estimates of resource investment in sexual reproduction (i.e., seed number, floret number, seed-setting rate, seed biomass, and panicle biomass) and the number and biomass of vegetative ramets connected to tillering nodes in natural populations of *H. glabra* (Figure 1A–E and Figure 3A–E). These results are different from those previously found in clonal grasses and indicate that *H. glabra* could increase floret and seed production and simultaneously produce more vegetative ramets. It has been reported that a high level of integration between connected ramets in clonal plants may obscure the mutual limitation of the investment of resources occurring between clonal reproduction and sexual reproduction [50]. Therefore, our results may imply that there is a high degree of physiological integration between different functional ramets connected by tillering nodes in the natural populations of *H. glabra*.

Physiological integration is one of the distinctive life history strategies of clonal plants, and ramets can transfer and share resources through physical connections [5,6]. Stable isotope labeling is one of the effective methods to verify resource translocation between clonal ramets [9]. Although a study regarding clonal grass *Agrostis stolonifera* illustrated the necessity of bidirectional tracing of resource translocation to estimate net flows of resources in clonal systems [9], it focused only on resource translocation between vegetative ramets and vegetative ramets with the same function. In the present study, we focused on resource translocation between ramets with different functions, namely, vegetative ramets and reproductive ramets. When the leaves of vegetative ramets were labeled with ^15^N at the seed-filling stage of *H. glabra*, a significantly larger amount of ^15^N than the background value was detected in various organs of the connected reproductive ramets (Figure 4), demonstrating that vegetative ramets could supply resources to reproductive ramets through physiological integration at the critical stage of sexual reproduction. There are two major reasons for conducting monodirectional tracing of resource translocation between different functional ramets in the natural populations of *H. glabra*. On the one hand, we found that reproductive ramets usually had two leaves (Table 1), and at the seed-filling stage, the second leaf (from the top of the ramet) was almost completely withered and yellow; the first leaf was very soft and small, the leaf area was only 0.20 cm^2^ (Table 1), and more than 60% of the leaf surface was also withered and yellow. Therefore, the leaf activity of reproductive ramets should be extremely low. However, vegetative ramets had more and larger green leaves (Table 1) and thereby had larger leaf activity and photosynthetic potential than reproductive ramets. On the other hand, according to source-sink theory, once reproductive ramets begin reproductive growth, continuous apical dominance appears [51], and in particular, reproductive ramets have the greatest demand for resources at the seed-filling stage. Many studies have shown that the net direction of resource translocation may be determined by the location of the greatest demand for resources [52,53]. By this token, at the seed-filling stage of *H. glabra*, the net direction of resources translocation should be from vegetative ramets towards reproductive ramets in the ramet pairs composed of vegetative ramets and reproductive ramets.

Physiological integration can allow older ramets to support younger, developing ramets and permit ramets growing in high-resource patches to support ramets growing in low-resource patches [3,4]. In the present study, taking 2019 as an example, we found that the seed number, floret number, seed-setting rate, seed biomass, and panicle biomass of the reproductive ramets connected to three vegetative ramets were 10.8 times, 1.2 times, 9.0 times, 6.9 times, and 1.3 times, respectively, as great as those of the reproductive ramets connected to zero vegetative ramets (Figure 1A–E), indicating that the larger the number of vegetative ramets connected to the reproductive ramet was, the better the sexual reproductive performance was. More interestingly, we found that there was no significant difference in the biomass of reproductive ramets among different numbers of connecting vegetative ramets (Figure 1F), demonstrating that the number of vegetative ramets connected to tillering nodes had no effect on the overall growth of reproductive ramets. However, the biomass of reproductive ramets was found significantly and negatively affected by the soil moisture (Table 3). This is because, under high-water conditions, *H. glabra* may preferentially invest resources in the belowground rhizomes and buds for vegetative propagation [54], thereby limiting the overall growth of reproductive ramets. Considering the small density of ramets at the edge of the clone, the large interval between ramets, and the proximity of resource supply, compared with vegetative ramets on the tillering nodes, other vegetative ramets had tiny effects on sexual reproduction performance. Therefore, there is no doubt that physiological integration between vegetative ramets and reproductive ramets connected by tillering nodes can increase the seed production performance, which is beneficial to population persistence in *H. glabra*.

### 3.3. The Causes of H. glabra Being a Companion Species Rather than a Dominant Species in the Climax Plant Community

The division of dominant and companion species depends on differences in their status and role in the community, which are caused by their different biological and ecological characteristics. In semiarid and arid grassland plant communities in eastern Eurasia, it is generally acknowledged that perennial *L. chinensis* is a major dominant species and perennial *H. glabra* is a major companion species [29]. *H. glabra* is a typical early spring grass, with its seed maturing in early June and approximately one and a half months earlier than *L. chinensis*. In the present study, in the natural populations of *H. glabra*, we found that vegetative ramets connected to tillering nodes could translocate their own resources to reproductive ramets through tillering nodes for seed production, which is highly consistent with the findings in *L. chinensis* [55]. This implies that there is an evident commonality between *H. glabra* and *L. chinensis*; namely, during sexual reproduction, there is the physiological integration between different functional ramets connected by tillering nodes.

In this study, in the natural populations of *H. glabra* growing in relatively homogenous sandy soil (Supplementary Table S7), we found that a reproductive ramet connected to two vegetative ramets had an average of 1.2 more seeds and 0.9% greater seed-setting rate than that connected to one vegetative ramet in 2018 (Figure 1A,C). In the same year, our research group investigated the sexual reproductive characteristics of *L. chinensis* growing in homogenous sandy soil in this study area and found that a reproductive ramet connected to two vegetative ramets had an average of 2.5 more seeds and 5.2% greater seed-setting rate than that connected to one vegetative ramet [55]. By this token, *H. glabra* had a lower capacity for physiological integration than *L. chinensis*. The lower capacity for physiological integration allows vegetative ramets of *H. glabra* to translocate fewer resources to reproductive ramets, further affecting sexual reproduction and resulting in a lower seed-setting rate and fewer seeds, which is unfavorable for population persistence. Our study reveals for the first time that *H. glabra* can exist only as a companion species in plant communities, from the perspective of the effects of physiological integration on sexual reproduction.

## 4. Materials and Methods

### 4.1. Study Area

This study was carried out at the Grassland Ecological Research Station of Northeast Normal University, Jilin Province, China (44°38′ N, 123°41′ E) in 2018 and 2019. The study area is located in the eastern part of the Eurasian steppe and has a typical temperate monsoon climate, with rainy, hot summers and dry, cold winters. The annual mean temperature ranges from 4.6 °C to 6.4 °C, and the annual mean precipitation varies from 300 mm to 450 mm. The frost-free period lasts approximately 130–165 days [55].

### 4.2. Study Species

*Hierochloe glabra* (Trin.) is a perennial rhizomatous clonal plant that is widely distributed in the eastern part of the Eurasian steppe [56]. *H. glabra* has a strong tillering ability in addition to its slender and fleshy belowground rhizomes. Among the multiple ramets formed on each tillering node, there is usually only one reproductive ramet, and the rest are vegetative ramets. *H. glabra* can achieve population regeneration through both the vegetative propagation of rhizomes and tillering nodes and sexual reproduction via seeds in natural grasslands [57]. Because of its good palatability and high nutritional value, *H. glabra* is often used as a forage grass.

In this study area, *H. glabra* usually begins returning green and heading in late April, flowering in early May, seed maturing in early June [58], and exhibiting post-fruit rapid vegetative growth from early June to the end of September; the aboveground ramets wither, and the belowground rhizomes enter a dormant state in early October [30]. At the beginning of the returning green stage in the next year, the growth of aboveground ramets requires resource supply from belowground rhizomes until the leaves of ramets are fully expanded for photosynthesis [59].

### 4.3. Experimental Design

The study site was originally agricultural land, and the time since abandonment was more than ten years. It is currently in the process of natural restoration and plant community succession, and there are many *H. glabra* clones with different shapes and clear boundaries, and some other plant species, including *Setaria viridis*, *Chloris virgata*, *Tournefortia sibirica*, and *Chenopodium aristatum*. We selected twenty-five *H. glabra* clones of similar shapes (approximately circle) and similar sizes to carry out all the experiments at the population level. The adjacent clones were at least 5 m apart. The soil type was sandy soil [41,55].

### 4.4. Morphological Characteristics and Biomass Allocation

To investigate the difference in morphological characteristics and biomass allocation between reproductive and vegetative ramets of *H. glabra*, one ramet pair, consisting of one reproductive ramet and one vegetative ramet connected by a tillering node, was randomly collected at the edge of each clone at the seed-maturation stage in early June 2019. The length, width, and area of all leaves of each ramet were measured with a portable leaf area meter (AM350, ADC BioScientific Ltd., Herts, UK). The height of each ramet was measured. Each vegetative ramet was sorted into the leaves and sheath, while each reproductive ramet was separated into the leaves, sheath, stem, and panicle. The dry weight of all parts of each ramet was measured after oven-drying at 65 °C for 48 h.

### 4.5. Sexual Reproductive Characteristics

**Figure 5:**
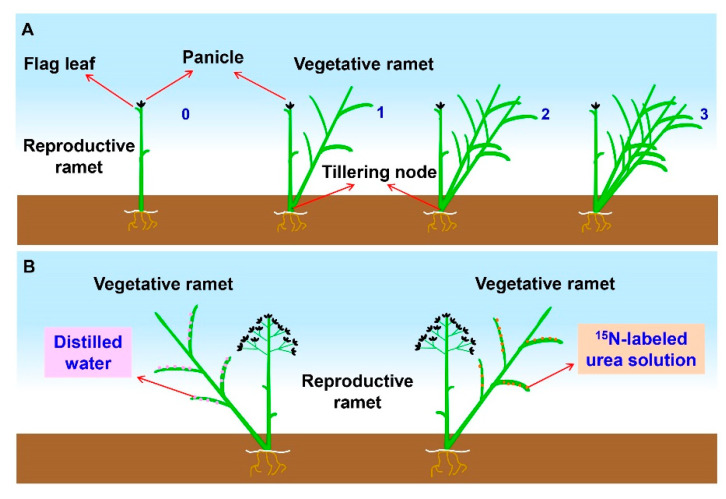
Schematic of the experiment design. (**A**) Schematic representation of the tagging manipulation at the early heading stage of *Hierochloe glabra* (the Arabic numerals represent the number of vegetative ramets connected to the reproductive ramet by tillering nodes. In each gradient, the panicle top of the reproductive ramet reaches approximately 2 cm over the flag leaf sheath). (**B**) Schematic representation of the stable-isotope (^15^N) labeling experimental design at the seed-filling stage of *Hierochloe glabra*. Each ramet pair consists of one reproductive ramet and one vegetative ramet connected by a tillering node.

To assess the effects of the number of vegetative ramets on sexual reproductive performance, we conducted a gradient (here refers to a graded change in the number of vegetative ramets connected to the tillering nodes of reproductive ramets) samplings in both 2018 and 2019. First, at the early heading stage of *H. glabra* (late April) in each year, one quadrat of 0.5 m × 0.5 m was fixed at the edge of each clone. In each quadrat, we used colored tags to mark reproductive ramets whose panicle top reached approximately 2 cm over the flag leaf sheath, and they were connected to different numbers (0, 1, and 2 in 2018; 0, 1, 2, and 3 in 2019) of vegetative ramets by tillering nodes (Figure 5A), and there was only one reproductive ramet per gradient in each quadrat. All marked reproductive ramets, together with their connected vegetative ramets, were harvested at the seed-maturation stage. The seed number and floret number were determined. Each reproductive ramet was sorted into leaves, stems, panicles, and seeds. After oven-drying at 65 °C for 48 h, the seed biomass, panicle biomass, reproductive ramet biomass, leaf biomass, and total biomass of the connected vegetative ramets were measured.

### 4.6. Physicochemical Properties of the Soil

In 2018, after the gradient samplings of *H. glabra*, soils were sampled from the top 15 cm at five randomly distributed locations within each quadrat (0.5 m × 0.5 m) using a soil corer (2.54 cm in diameter). Five soil cores were collected and then mixed into one single sample within each quadrat. Soil moisture was determined with the gravimetric method by weighing soil samples after oven-drying at 105 °C for 48 h. Soil bulk density was estimated by the W/V method (W is the soil weight in grams after oven-drying at 105 °C for 48 h, and V is 100 cm^3^) [60]. Soil pH and electric conductivity were determined using sediment slurries (10 g air-dried soil samples and 50 mL deionized water were shaken for 1 h, 250 rpm) [61]. Soil organic C was measured by the K_2_Cr_2_O_7_ titration method after digestion [62]. Soil total N was measured using a Kjeltec 2300 analyzer unit (FOSS, Hilleroed, Denmark) after digestion [63]. Soil total P was determined with the HClO_4_-H_2_SO_4_ digestion method [64].

### 4.7. Stable Isotope Labeling

To verify whether vegetative ramets translocate their own resource to the connected reproductive ramets during sexual reproduction, an in situ-leaf labeling experiment was carried out at the seed-filling stage of *H. glabra* in mid-May 2019. We randomly selected five out of twenty-five clones to carry out the stable isotope labeling experiment. One quadrat of 1 m × 1 m was randomly fixed at the edge of each clone. Two ramet pairs of similar sizes (one ramet pair for the control treatment and another for the ^15^N labeling treatment) were chosen in each quadrat, and each ramet pair consisted of one vegetative ramet (daughter ramet) and one reproductive ramet (mother ramet) connected by the tillering node (Figure 5B). The two ramet pairs were at least 50 cm apart.

To avoid soil contamination, ^15^N labeling was carried out during a succession of sunny days, and therefore, the ^15^N label on the leaves of ramets would not be washed to the soil; in addition, the soil surface was tightly covered with plastic wrap before labeling. Then, 1 mL of the ^15^N-labeled urea (made at the Shanghai Research Institute of Chemical Industry, China) solution with a ^15^N abundance of 5.18% and a urea concentration of 0.02 g·mL^−1^ was applied to the vegetative ramet of each ramet pair following the protocol from Guo et al. [55]. Labeling was implemented once per day for three consecutive days. An equal volume of distilled water was used instead of the ^15^N-labeled urea solution in the control treatment. The aboveground reproductive ramets were harvested exactly 2 days after labeling and sorted into the leaves, stems (including the sheath), and panicles. All samples were then dried (65 °C for 48 h) and ground to a fine powder with a ball mill (MM 400 Retsch, Haan, Germany) before the isotopic analyses. The isotope values (*δ*^15^N) of all samples were determined using a vario EL cube elemental analyzer (Elementar, Langenselbold, Germany) interfaced to an Isoprime 100 isotope-ratio mass spectrometer (Elementar, Langenselbold, Germany).

### 4.8. Statistical Analysis

All statistical analyses were carried out using SPSS software version 22.0 (SPSS Inc., Chicago, IL, USA). All data were tested for adherence to a normal distribution and homogeneous variances through a Kolmogorov–Smirnov test and Levene’s test, respectively. Descriptive statistics were used to analyze the physicochemical properties of the soil. An independent samples *t*-test was used to determine the differences in height, total leaf area, and total biomass between reproductive and vegetative ramets; to assess the differences in leaf length, width, and the area between the two leaves of reproductive ramets; to determine the differences in biomass between the two organs of vegetative ramets; to test the differences in leaf *δ*^15^N, stem *δ*^15^N, and panicle *δ*^15^N between the control and ^15^N labeling treatments. One-way analysis of variance (ANOVA) was used to determine the effect of leaf position on the leaf length, width, and area of vegetative ramets; to assess the effect of organ type on the biomass and biomass allocation of reproductive ramets; to determine the effect of the number of connected vegetative ramets on sexual reproductive performance. Duncan’s multiple range test was used to test for significant differences between the means of multiple groups. The sexual reproductive performance indexes included seed number, floret number, seed-setting rate (seed number relative to floret number, expressed as a percentage), seed biomass, panicle biomass, and reproductive ramet biomass.

Pearson correlation analyses were used to investigate the relationships between soil physicochemical properties and sexual reproductive performance as well as the growth characteristics of vegetative ramets. To reveal the change rules of sexual reproductive performance with respect to the differences in the leaf biomass or total biomass of the connected vegetative ramets, the six sexual reproductive indexes were regressed on the difference in the leaf biomass or total biomass of connected vegetative ramets using linear, exponential, power, and logarithmic functions in both years, and the model with the lowest Akaike information criterion (AIC) value from these four was finally considered the best-fitting model [65].

## 5. Conclusions

In the natural populations of the rhizomatous grass *H. glabra*, vegetative ramets were taller and had more leaves with a larger area and had a greater biomass than reproductive ramets. The physicochemical properties of the soil basically had no significant effect on sexual reproductive performance. However, during sexual reproduction, vegetative ramets connected by tillering nodes to reproductive ramets could transfer their own nutrients to the reproductive ramets through physiological integration, thus significantly increasing sexual reproductive performance (i.e., seed number, floret number, seed-setting rate, seed biomass, and panicle biomass), which would be beneficial to population persistence in *H. glabra*.

## Figures and Tables

**Table 1:** Morphological characteristics of reproductive ramets and vegetative ramets in the natural populations of *Hierochloe glabra* (data are represented as the means ± standard errors, *n* = 25).

Characteristic	Type	Reproductive Ramet	Vegetative Ramet
Ramet height (cm)		32.66 ± 0.86	52.86 ± 0.67 ***
Leaf length (cm)	1st leaf	1.32 ± 0.11 b	35.57 ± 0.57 a
	2nd leaf	1.94 ± 0.12 a	26.85 ± 0.53 b
	3rd leaf		22.09 ± 0.47 c
	4th leaf		16.41 ± 0.64 d
Leaf width (cm)	1st leaf	0.26 ± 0.02 b	0.93 ± 0.01 a
	2nd leaf	0.36 ± 0.01 a	0.96 ± 0.02 a
	3rd leaf		0.87 ± 0.02 b
	4th leaf		0.65 ± 0.02 c
Leaf area (cm^2^)	1st leaf	0.20 ± 0.03 b	26.41 ± 0.83 a
	2nd leaf	0.43 ± 0.04 a	22.06 ± 0.53 b
	3rd leaf		16.58 ± 0.71 c
	4th leaf		9.45 ± 0.55 d
	Total	0.63 ± 0.06	74.50 ± 1.98 ***

Note: the leaf position is from the top to the base of a ramet; different lowercase letters for each characteristic in each type of ramet indicate significant differences among the leaves (*p* < 0.05); *** indicates significant differences between the two types of ramets (*p* < 0.001).

**Table 2:** Mean biomass allocation to various organs of reproductive ramets and vegetative ramets in the natural populations of *Hierochloe glabra* (data are represented as the means ± standard errors, *n* = 25).

Organ	Reproductive Ramet		Vegetative Ramet	
	Biomass (mg)	Percentage (%)	Biomass (mg)	Percentage (%)
Leaf	16.12 ± 1.02 c	7.88 ± 0.48 d	467.00 ± 13.87 a	61.32 ± 0.72 a
Sheath	57.16 ± 2.49 b	27.52 ± 0.63 c	294.84 ± 9.92 b	38.68 ± 0.72 b
Stem	62.88 ± 2.75 b	30.31 ± 0.75 b		
Panicle	70.72 ± 2.58 a	34.29 ± 0.69 a		
Total	206.88 ± 6.81	100.00	761.84 ± 21.34 ***	100.00

Note: different lowercase letters for the same type of ramet indicate significant differences among the organs (*p* < 0.05); *** indicates significant differences between the two different types of ramets (*p* < 0.001).

**Table 3:** Relationships between soil physicochemical properties and sexual reproductive characteristics as well as growth characteristics of vegetative ramets in the natural populations of *Hierochloe glabra* in 2018. Pearson’s correlation coefficients are shown.

Characteristic	Moisture	Bulk Density	pH	Electric Conductivity	Total Organic C	Total N	Total P
Seed number	−0.052	−0.055	−0.092	−0.040	0.014	−0.083	0.036
Floret number	−0.085	−0.142	−0.023	−0.006	0.036	−0.042	−0.088
Seed-setting rate	−0.013	−0.025	−0.023	−0.018	−0.003	−0.074	0.067
Seed biomass	−0.047	−0.019	−0.075	−0.083	0.066	0.003	−0.008
Panicle biomass	−0.152	−0.152	−0.076	0.072	0.075	−0.040	−0.041
Reproductive ramet biomass	−0.247 *	−0.068	−0.119	0.094	0.050	−0.027	−0.025
Leaf biomass of vegetative ramet	−0.022	−0.058	−0.070	−0.076	0.099	−0.033	−0.136
Vegetative ramet biomass	−0.006	−0.061	−0.061	−0.060	0.081	−0.026	−0.132

Note: *, *p* < 0.05.

## Data Availability

The datasets generated during and/or analyzed during the current study are available from the corresponding author on reasonable request.

## Supplementary Materials

The following are available online at https://www.mdpi.com/2223-7747/9/11/1608/s1, Table S1: Results of independent samples *t*-test between reproductive ramets and vegetative ramets in the natural populations of *Hierochloe glabra* (*n* = 25), Table S2: Results of independent samples *t*-test between 1st leaf and 2nd leaf of reproductive ramets in the natural populations of *Hierochloe glabra* (*n* = 25), Table S3: Results of one-way ANOVA testing the effect of leaf position on the leaf length, width, and area of vegetative ramets in the natural populations of *Hierochloe glabra* (*n* = 25), Table S4: Results of one-way ANOVA testing the effect of organ type on biomass and allocation percentage of reproductive ramets in the natural populations of *Hierochloe glabra* (*n* = 25), Table S5: Results of independent samples *t*-test between leaf biomass (allocation percentage) and sheath biomass (allocation percentage) of vegetative ramets in the natural populations of *Hierochloe glabra* (*n* = 25), Table S6: Results of one-way ANOVA testing the effect of the number of connected vegetative ramets on sexual reproductive performance in the natural populations of *Hierochloe glabra* (*n* = 25), Table S7: Physicochemical properties of the soil in the twenty-five clones of *Hierochloe glabra*, Table S8: Results of independent samples *t*-test between the control and ^15^N labeling treatments in the natural populations of *Hierochloe glabra* (*n* = 5).
