# Supplementary material for: Physiological Integration Increases Sexual Reproductive Performance of the Rhizomatous Grass Hierochloe glabra
Source: Plants (Basel). 2020 Nov 19;9(11):1608. doi: 10.3390/plants9111608 (PMC7699368; doi:10.3390/plants9111608)
Supplement: Supplementary file 1 [file plants-09-01608-s001.pdf]

plants

MDPI

Article

Physiological integration increases sexual reproductive performance of the rhizomatous grass *Hierochloe glabra*

Jian Guo, Haiyan Li * and Yunfei Yang *

Key Laboratory of Vegetation Ecology, Ministry of Education, Institute of Grassland Science, Northeast Normal University, Jilin, Changchun 130024, China; guoj481@nenu.edu.cn (J.G.)

* Correspondence: lihy697@nenu.edu.cn (H.L.); yangyf@nenu.edu.cn (Y.Y.)

Received: date; Accepted: date; Published: date

Supplementary Materials

Supplementary Table S1 Results of independent-samples *t*-test between reproductive ramets and vegetative ramets in the natural populations of *Hierochloe glabra* (n = 25).

Characteristic	t	p
Ramet height	-18.52	< 0.001
Total area	-37.36	< 0.001
Total biomass	-24.78	< 0.001

Supplementary Table S2 Results of independent-samples *t*-test between 1st leaf and 2nd leaf of reproductive ramets in the natural populations of *Hierochloe glabra* (n = 25).

Characteristic	t	p
Leaf length	-3.73	0.001
Leaf width	-5.32	< 0.001
Leaf area	-5.09	< 0.001

Supplementary Table S3 Results of one-way ANOVA testing the effect of leaf position on the leaf length, width and area of vegetative ramets in the natural populations of *Hierochloe glabra* (n = 25).

Characteristic	$F_{3,96}$	p
Leaf length	212.28	< 0.001
Leaf width	45.22	< 0.001
Leaf area	120.34	< 0.001

Supplementary Table S4 Results of one-way ANOVA testing the effect of organ type on biomass and allocation percentage of reproductive ramets in the natural populations of *Hierochloe glabra* (n = 25).

Characteristic	$F_{3,96}$	p
Biomass	110.60	< 0.001

Allocation percentage	329.94	< 0.001

Supplementary Table S5 Results of independent-samples *t*-test between leaf biomass (allocation percentage) and sheath biomass (allocation percentage) of vegetative ramets in the natural populations of *Hierochloe glabra* ($n = 25$).

**Characteristic**	t	p
Biomass	10.09	< 0.001
Allocation percentage	22.23	< 0.001

Supplementary Table S6 Results of one-way ANOVA testing the effect of the number of connected vegetative ramets on sexual reproductive performance in the natural populations of *Hierochloe glabra* ($n = 25$).

**Characteristic**	**2018**		**2019**	
	$F_{2,72}$	p	$F_{3,96}$	p
Seed number	24.72	< 0.001	16.08	< 0.001
Floret number	14.60	< 0.001	8.95	< 0.001
Seed-setting rate	18.25	< 0.001	14.71	< 0.001
Seed biomass	24.04	< 0.001	10.89	< 0.001
Panicle biomass	7.41	0.001	9.30	< 0.001
Ramet biomass	0.84	0.436	0.18	0.912

Supplementary Table S7 Physicochemical properties of the soil in the twenty-five clones of *Hierochloe glabra*.

**Clone**	**Moisture (%)**	**Bulk density ($g\ cm^{-3}$)**	**pH**	**Electric Conductivity ($\mu S\ cm^{-1}$)**	**Total organic C ($g\ kg^{-1}$)**	**Total N ($g\ kg^{-1}$)**	**Total P ($g\ kg^{-1}$)**
1	10.52	1.25	8.30	73.00	5.86	1.01	0.74
2	12.07	1.11	8.38	74.90	6.02	1.04	0.80
3	11.31	1.20	8.44	77.70	5.86	1.06	0.73
4	11.56	1.10	8.46	78.80	5.10	1.03	0.79
5	10.90	1.12	8.11	73.00	5.82	1.05	0.75
6	12.03	1.22	8.35	73.10	5.64	1.05	0.79
7	10.95	1.26	8.40	75.10	6.00	1.02	0.76
8	11.42	1.20	8.17	71.80	5.67	1.06	0.74
9	11.57	1.19	8.30	72.80	5.86	1.03	0.76
10	10.60	1.17	8.20	73.90	5.17	1.03	0.74
11	11.46	1.22	8.33	74.00	5.24	1.06	0.79
12	11.00	1.18	8.25	75.80	5.27	1.02	0.76
13	10.64	1.20	8.41	70.90	6.36	1.03	0.72
14	11.58	1.22	8.18	73.10	6.19	1.05	0.75
15	11.92	1.24	8.49	78.30	5.41	1.03	0.73
16	10.78	1.24	8.35	74.30	5.51	1.06	0.76
17	11.23	1.13	8.43	76.80	5.55	1.06	0.75
18	11.48	1.24	8.19	78.00	6.16	1.03	0.78

19	11.60	1.13	8.28	71.20	6.12	1.05	0.75
20	11.74	1.21	8.45	70.00	5.68	1.03	0.73
21	10.88	1.12	8.35	74.30	5.82	1.08	0.73
22	11.29	1.16	8.24	72.80	5.85	1.05	0.76
23	11.54	1.28	8.46	76.70	5.85	1.04	0.74
24	11.61	1.29	8.48	71.50	6.12	1.04	0.78
25	11.63	1.12	8.18	77.60	5.92	1.06	0.74

Supplementary Table S8 Results of independent-samples *t*-test between the control and ^{15}N labeling treatments in the natural populations of *Hierochloe glabra* ($n = 5$).

Characteristic	t	p
Leaf $\delta^{15}N$	-14.42	< 0.001
Stem $\delta^{15}N$	-30.96	< 0.001
Panicle $\delta^{15}N$	-13.25	< 0.001

© 2020 by the authors. Submitted for possible open access publication under the terms and conditions of the Creative Commons Attribution (CC BY) license (http://creativecommons.org/licenses/by/4.0/).
